# Walking the tightrope: Mixed respiratory failure exacerbated by heart rate control and concurrent pneumonia in an elderly patient with new-onset atrial fibrillation: A case report

**DOI:** 10.1097/MD.0000000000050388

**Published:** 2026-08-21

**Authors:** Yulong Huang, Haodong Huang, Hong Yan, Xin Zhang, Fangting Wang, Yujing Chen, Zhitong Deng, Xiaojun Yang

**Affiliations:** a Haikou Hospital of Traditional Chinese Medicine, Haikou, Hainan, China; b First Affiliated Hospital of Guangzhou University of Chinese Medicine, Guangzhou, Guangdong, China.

**Keywords:** atrial fibrillation, case report, geriatric cardiology, heart failure with preserved ejection fraction (HFpEF), mixed respiratory failure, pleural effusion, rate control

## Abstract

**Rationale:**

The interplay between new-onset atrial fibrillation (AF), heart failure (HF) with preserved ejection fraction (HFpEF), and acute infection in the elderly presents a complex clinical challenge. Rate control is a cornerstone of AF management, but its application in frail patients with limited cardiac reserve and concurrent infection remains uncertain. This case illustrates the diagnostic and therapeutic difficulties encountered when these conditions converge, highlighting the need for dynamic assessment and individualized treatment strategies.

**Patient concerns:**

An 86-year-old woman with a history of hypertension, HFpEF, and cerebral infarction presented with a 3-day history of worsening dizziness and dyspnea. On admission, she was afebrile with clear lung auscultation but had a rapid, irregular heart rate of 152 bpm.

**Diagnoses:**

Electrocardiogram confirmed new-onset AF with rapid ventricular response. Elevated N-terminal pro-B-type natriuretic peptide confirmed acute HF exacerbation. Although an initial chest computed tomography showed only minor chronic changes, the patient subsequently developed fever, productive cough, and hypoxemia. A repeat chest computed tomography revealed new multifocal pulmonary infiltrates and bilateral pleural effusions. Sputum culture later confirmed *Klebsiella pneumoniae* infection. The final diagnosis was mixed respiratory failure exacerbated by rapid AF in a patient with underlying HFpEF.

**Interventions:**

Initial treatment included diuresis, anticoagulation, and rate control with metoprolol tartrate. Upon clinical deterioration, noninvasive ventilation was initiated. Anti-infective therapy was intensified empirically with moxifloxacin and ceftriaxone, later adjusted to ceftriaxone monotherapy based on culture results and an adverse reaction to moxifloxacin. Diuretic therapy was continued, and the heart rate target was relaxed to 80 to 100 bpm. Electrolyte and acid-base status were closely monitored and corrected.

**Outcomes:**

The patient’s hemodynamics stabilized, respiratory status improved, and N-terminal pro-B-type natriuretic peptide declined, with pleural effusions largely resolved on ultrasound. She was discharged home in stable condition after a 14-day hospital stay on guideline-directed medical therapy.

**Lessons:**

In geriatric patients, infection can present atypically and act as a silent trigger for HF decompensation, leading to mixed respiratory failure. Rate control targets should be individualized and dynamically adjusted, particularly during acute illness, to avoid potential compromise of cardiac output. This case underscores the importance of diagnostic vigilance, serial imaging, electrolyte monitoring, and flexible, pathophysiology-guided management in optimizing outcomes in this vulnerable population.

## Graphical abstract

**Figure:**
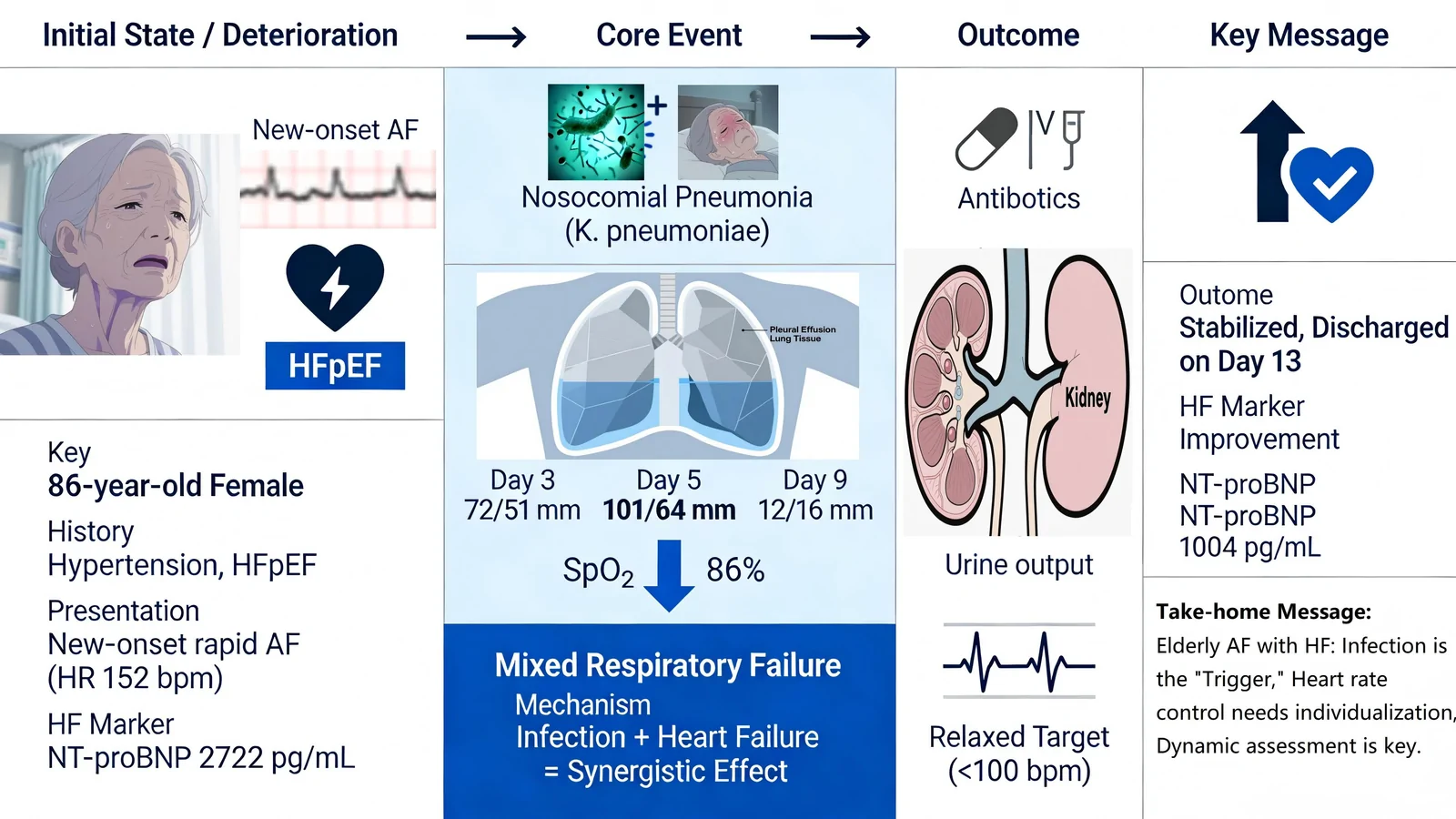

## 1. Introduction

Atrial fibrillation (AF) and heart failure (HF) frequently coexist, creating a detrimental cycle.^[1]^ The prevalence of AF increases markedly with age, posing a particularly significant health burden in octogenarians and beyond. In these vulnerable populations, AF is independently associated with heightened risks of thromboembolic stroke, HF hospitalization, cognitive decline, and all-cause mortality.^[2,3]^ The 2023 European Society of Cardiology Guidelines for the management of AF emphasize rate control as a fundamental strategy.^[4]^ However, in patients with concomitant HF, particularly those with HF with preserved ejection fraction (HFpEF), cardiac reserve is often limited, and cardiac output is highly dependent on preload and heart rate.^[5,6]^ In this context, the relationship between heart rate reduction and hemodynamic decompensation is complex and not fully established. Aggressive rate control may theoretically compromise cardiac output, but whether this translates into clinical deterioration in frail elderly patients remains uncertain, and current evidence is largely hypothesis-generating rather than conclusive. The situation becomes more complex when infection is superimposed. Infection can both precipitate AF^[7]^ and directly worsen cardiac function,^[8]^ acting as a “trigger” for acute HF decompensation. Other common precipitating factors for AF in the elderly include hypertension, structural heart disease, inflammation, malnutrition, and age-related atrial remodeling, all of which contribute to the vulnerable substrate in this population.^[9,10]^ The case we report offers an opportunity to examine the difficulty of navigating rate control amidst the complexities of infection and HF, while acknowledging that multiple factors likely contributed to the clinical course. It reinforces the principles of dynamic assessment and individualized medicine in complex geriatric cardiology.

## 2. Case presentation

### 2.1. Patient information

An 86-year-old Chinese woman. Past medical history: Grade 3 hypertension, hypertensive heart disease, HFpEF (prior echocardiogram showed left atrial enlargement, diastolic dysfunction, and preserved left ventricular ejection fraction [LVEF]), cerebral infarction, hyperlipidemia, and osteoporosis.

### 2.2. Chief complaint and history of present illness

The patient presented with a 3-day history of worsening dizziness and shortness of breath, against a background of over 14 years of hypertension-related dizziness. She reported no chest pain, fever, or productive cough at the time of admission.

### 2.3. Physical examination on admission

Vital signs: blood pressure 131/89 mm Hg, heart rate 152 bpm (irregular), respiratory rate 20 bpm, temperature 36.5°C. Cardiac examination revealed an irregularly irregular rhythm with variable first heart sound and a pulse deficit, consistent with AF. Lung auscultation was clear bilaterally with no adventitious sounds.

### 2.4. Diagnostic assessment

#### 2.4.1. Laboratory tests

N-terminal proB-type natriuretic peptide (NT-proBNP) was markedly elevated at 2722 pg/mL (reference < 125 pg/mL), confirming acute HF. Troponin I was mildly elevated (0.061 ng/mL). Complete blood count and high-sensitivity C-reactive protein were within normal limits. Admission serum potassium was 3.89 mmol/L (reference range 3.5–5.3 mmol/L).

#### 2.4.2. Electrocardiogram (ECG)

**Figure 1.**
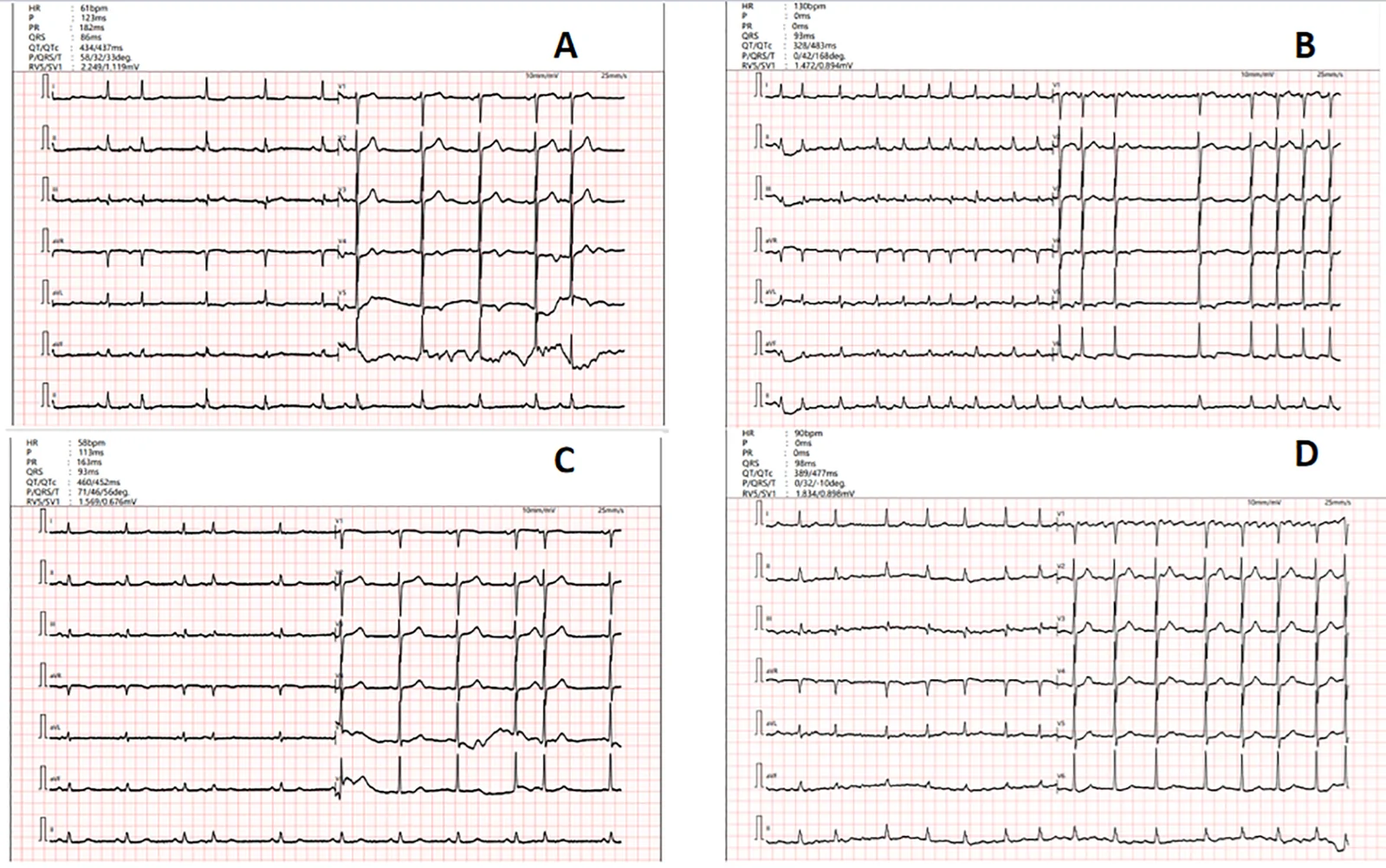
Serial ECGs and clinical course. (A) ECG 1 month prior to admission: shows sinus rhythm, no AF. (B) ECG on admission: shows new-onset AF with a rapid ventricular rate (approximately 152 bpm). (C) ECG on the third day of admission: shows AF with a slow ventricular rate (approximately 58 bpm). (D) ECG at discharge: shows rate-controlled AF. AF = atrial fibrillation, ECG = electrocardiogram,

Compared to a sinus rhythm ECG from 1 month prior (Fig. 1A), the admission ECG showed new-onset AF with a rapid ventricular rate (152 bpm) (Fig. 1B).

#### 2.4.3. Imaging

**Figure 2.**
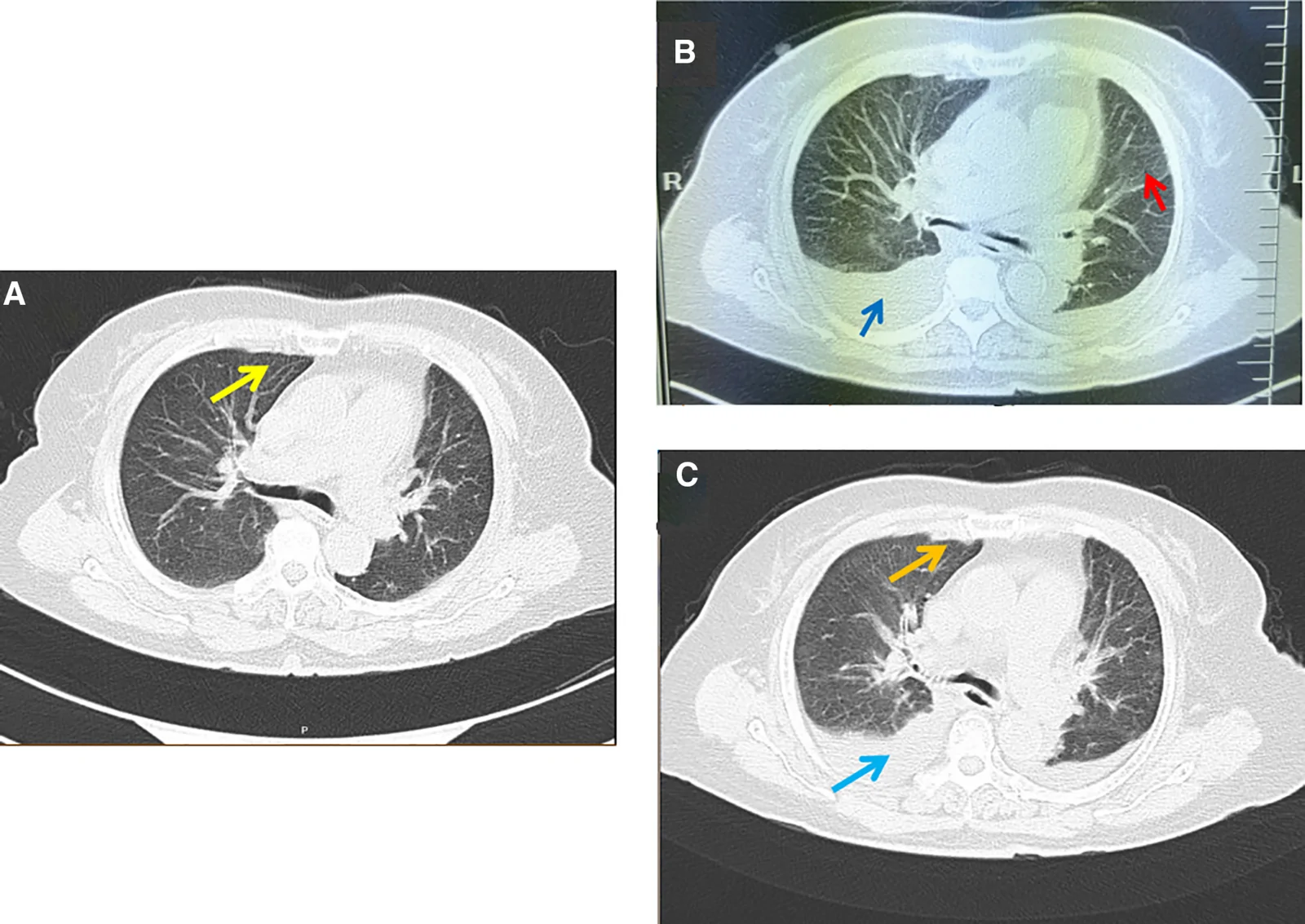
Lung CT and clinical course. (A) Chest CT obtained prior to admission, showing mild inflammatory lesions in the lungs (as indicated by the yellow arrow). (B) and (C) Chest CT performed on the third day of hospitalization, revealing multifocal inflammatory lesions in both lungs (left-sided lung infections are marked with red arrows, and right-sided lung infections are marked with orange arrows) and pleural effusion (as indicated by the blue arrow). CT = computed tomography.

An echocardiogram revealed left atrial enlargement, diastolic dysfunction, and preserved LVEF, consistent with HFpEF. A chest computed tomography scan showed minor chronic inflammatory changes in both lungs (Fig. 2A).

### 2.5. Hospital course and therapeutic interventions

The patient was admitted for acute HF exacerbation triggered by rapid AF. Initial treatment included diuresis (furosemide, 20 mg, intravenously, when necessary), anticoagulation, and initiation of rate control with a beta-blocker (metoprolol tartrate, 25 mg, orally, twice daily). On hospital days 2 to 3, her condition took a turn. Although her heart rate was better controlled, she developed fever, cough, and sputum production, which rapidly progressed to tachypnea, orthopnea, and hypoxemia (peripheral capillary oxygen saturation 86%). Blood pressure transiently rose to 158/89 mm Hg. She required emergent noninvasive ventilation. An urgent chest computed tomography showed new multifocal inflammatory infiltrates (Fig. 2B) and bilateral pleural effusions, significantly worsened from admission. Subsequent bedside ultrasound monitoring captured the dynamic evolution of the effusion: on the 3rd day of admission, the maximum depth of the right pleural effusion was 72 mm, and the left was 51 mm; by the 5th day of admission, the effusion reached its peak, with the right side increasing to 101 mm and the left to 64 mm, accompanied by significant floating of the lung lobes (Fig. 2C). Following the intensification of anti-infective therapy (moxifloxacin hydrochloride, using pump intravenously 100 mL/hour, once daily) and diuresis, a follow-up ultrasound on the 9th day of admission showed that the effusion had largely resolved, with only 12 mm remaining on the right and 16 mm on the left (Table 1). This imaging improvement was highly synchronized with the patient’s clinical recovery and the decline in NT-proBNP levels (from a peak of 2722 pg/mL to 1004 pg/mL). Sputum culture later returned positive for *Klebsiella pneumoniae* (corresponding antibiotic sensitivity tests are shown in Table S1, Supplemental Digital Content 1).

**Table 1:** Key clinical events and multifactorial analysis.

Day of admission	Key event	NT-proBNP (pg/mL)	Oxygenation index (PaO_2_/FiO_2_)	Pleural effusion (mm, right, left)	Serum K^+^ (mmol/L)	Acid-base status (pCO_2_/ HCO_3_^−^)	Core analysis & intervention
1	Admission, new-onset rapid AF	2722	Not measured	-	3.89	-	Initiated HF and rate control therapy.
3	Peak illness: respiratory failure	1655	218 mm Hg	72/ 51	4.20	38/ 23.5	Mixed respiratory failure: synergistic effect of infection (*Klebsiella pneumoniae*) and HF. Empirical antibiotics initiated: ceftriaxone 2g iv qd (noon) and moxifloxacin 0.4g iv qd (evening). Emergent NIV, adjusted antibiotics and diuretics.
4	Deterioration	-	-	-	3.60	38/ 24.1	Continued clinical decline; hypokalemia noted, supplementation initiated.
5	Progression: maximal effusion	806	238 mm Hg	101/ 64	4.10	39/ 24.2	Infection peak; effusion volume reached maximum. Moxifloxacin discontinued due to neuropsychiatric adverse events; ceftriaxone monotherapy continued.
6	Clinical stability	-	-	-	3.70	39/ 27.1	Metabolic alkalosis trend emerging; clinical condition stabilizing.
9	Improvement: effusion resolving	-	Not measured	12/ 16	-	-	Effusion significantly decreased, reflecting effective decongestion and infection control. Cardiac function improved (NT-proBNP downtrend).
14	Stabilization, discharge	1004	Normal	-	3.76	-	Effective comprehensive therapy, improved cardiac function and infection control. Echocardiography: FS 33%, EF 62%.

AF = atrial fibrillation, bid = bis in die (twice daily), EF = ejection fraction, FiO2 = fraction of inspired oxygen, FS = fractional shortening, HF = heart failure, HFpEF = heart failure with preserved ejection fraction, iv = intravenosus (intravenously), NIV = noninvasive ventilation, NT-proBNP = N-terminal pro-B-type natriuretic peptide, PaO2 = partial pressure of oxygen, po = per os (orally), qd. = quaque die (once daily), SpO2 = peripheral capillary oxygen saturation.

Serial arterial blood gas measurements revealed an evolving acid-base status that paralleled the clinical course. On day 3, at the onset of respiratory failure, the pCO_2_ was 38 mm Hg and HCO_3_^−^ was 23.5 mmol/L, indicating a normal compensatory state. By day 6, as the patient’s condition progressed, the HCO_3_^−^ had risen to 27.1 mmol/L (with a pCO_2_ of 39 mm Hg), suggesting a trend toward metabolic alkalosis, likely reflecting a compensatory response to the ongoing infection and diuretic therapy. This trend partially reversed by day 11 (pCO_2_ 43 mm Hg, HCO_3_^−^ 24.9 mmol/L), corresponding with clinical stabilization. Serum potassium levels were closely monitored throughout. After an initial value of 3.89 mmol/L, potassium dipped to a nadir of 3.6 mmol/L on day 4, which coincided with the peak of diuretic therapy and the patient’s most unstable phase. This mild hypokalemia was corrected with supplementation, and levels remained stable thereafter (Table 1).

This clinical deterioration resulted from multiple interacting factors:

1. The “Trigger” of pulmonary infection: an acute exacerbation of a subclinical pulmonary infection triggered a systemic inflammatory response and hypoxia, significantly increasing cardiac workload.
2. The synergistic effect of HF and infection: The combination of infection and tachycardia (rapid AF) overwhelmed the already compromised heart, rapidly progressing to acute HF and pulmonary edema, ultimately manifesting as mixed respiratory failure.
3. The uncertain role of rate control: In the context of infection-induced hemodynamic instability, the initial rate control strategy represents a potential contributing factor, but an alternative explanation is that the rapid ventricular rate was itself a compensatory response to infection. The observed slowing of the heart rate (Fig. 1C) coincided with, but cannot be proven to have caused, the clinical deterioration. Both progression of pneumonia and worsening HF remain equally plausible drivers.

With the etiology clarified, the treatment strategy was promptly adjusted: antibiotics were intensified (guided by culture results), diuresis was continued to optimize volume status, and the heart rate target was relaxed to a more tolerable range (resting rate 80–100 bpm). Following these comprehensive adjustments, the patient’s hemodynamics stabilized (echocardiography: fractional shortening 33%, ejection fraction 62%), respiratory status improved, and NT-proBNP levels showed a downward trend to 1004 pg/mL.

On the evening of hospital day 5, the patient acutely developed altered mental status characterized by muttering to herself, incoherent speech, and difficulty initiating sleep. Given the temporal association with the administration of moxifloxacin (0.4 g intravenously once daily, initiated on the evening of day 3), these neuropsychiatric symptoms were considered to be related to the medication. Moxifloxacin was promptly discontinued. The antibiotic regimen was subsequently streamlined to ceftriaxone (2 g intravenously once daily, which had been initiated at noon on day 3), and this monotherapy was continued until day 12. Symptomatic management was initiated, including intravenous hydration and sedation with diazepam (5 mg, intramuscular). The patient subsequently became calm and fell asleep, with full resolution of the psychiatric disturbance. No further adverse events were observed during the remainder of the hospital stay.

### 2.6. Follow-up and outcome

The patient was discharged home in stable condition after a 14-day hospitalization (Fig. 1D), on guideline-directed medical therapy for HF, AF, and secondary prevention.

## 3. Discussion

This case offers several core teaching points for clinicians managing complex elderly patients with cardiovascular and respiratory comorbidities.

### 3.1. Diagnostic dynamism: the importance of vigilance in geriatric populations

The patient’s initial presentation (dominated by dizziness and dyspnea without fever or cough) could easily have been attributed solely to acute HF triggered by rapid AF. However, the subsequent deterioration highlighted a subclinical pulmonary infection that was not apparent on admission. This underscores a critical lesson: in elderly patients, atypical or blunted presentations of infection are common, and dynamic reassessment is essential. Reliance on initial impressions may delay recognition of superimposed pathology, particularly when physiological reserves are limited.

### 3.2. Mixed respiratory failure: a synergistic, not simply additive, process

This case vividly demonstrates how infection and HF interact synergistically to produce respiratory failure, a concept often underappreciated in clinical practice. Rather than attributing deterioration to a single cause, the clinician must recognize that infection acts as a “trigger,” while underlying heart disease provides the “pathological substrate” for rapid decompensation.

The temporal evolution of pleural effusion is consistent with this dual mechanism:

1. Peak effusion volume (day 5) coincided with the maximal inflammatory response, which may reflect infection-induced capillary leakage.
2. Rapid resolution (by day 9) followed effective decongestion and infection control, suggesting a cardiogenic contribution.

This imaging-clinical correlation may serve as a useful illustrative example: bedside ultrasound monitoring of pleural effusion can serve as a simple, noninvasive indicator of the “infection-HF” interaction and guide treatment titration.

### 3.3. The roles of acid-base balance and electrolytes: compensatory mechanisms and therapeutic targets

The serial blood gas and electrolyte data in this case provide valuable insight into the patient’s physiological response to acute illness and treatment. On day 3, at the onset of respiratory failure, the acid-base status was appropriately compensated (pCO_2_ 38 mm Hg, HCO_3_^−^ 23.5 mmol/L). However, by day 6, the development of a relative metabolic alkalosis (HCO_3_^−^ 27.1 mmol/L) was observed. This trend likely reflects a combination of factors: the metabolic compensation for any underlying respiratory acidosis, the effects of aggressive diuretic therapy (contraction alkalosis), and the ongoing inflammatory state. While mild, this shift underscores the complex interplay between the patient’s failing heart, the superimposed infection, and our therapeutic interventions.

Monitoring of serum potassium was particularly important in this case. Hypokalemia is a well-known risk factor for arrhythmias, including AF, and can be exacerbated by diuretic use.^[11]^ Our patient’s potassium level dropped to a nadir of 3.6 mmol/L on day 4, coinciding with the peak of diuretic therapy and the most unstable phase of her clinical course. This mild hypokalemia may have contributed to a lower threshold for arrhythmias and potentially influenced the response to rate control therapy. While it was promptly corrected, this episode highlights the importance of frequent electrolyte monitoring in such patients, as even mild disturbances can have significant electrophysiological consequences in the setting of acute AF and HF.^[12]^

### 3.4. Individualized heart rate targets: beyond guideline numbers

The 2023 European Society of Cardiology Guidelines recommend rate control in AF, but this case raises the possibility that rigid adherence to numerical targets could be hazardous in certain contexts. However, it is equally possible that the clinical deterioration was driven primarily by the infection itself, with rate control playing a neutral or even beneficial role. Aggressive beta-blockade might have limited compensatory capacity, but this remains speculative.

The key lesson is not to abandon rate control, but to recognize that in acutely ill patients with infection, rate targets should be individualized and frequently reassessed, as the optimal strategy is not defined by current evidence. A more lenient initial target (e.g., resting heart rate < 100–110 bpm) with subsequent titration after stabilization is a safer approach in this vulnerable population.

### 3.5. Holistic management: prioritizing the dominant pathology

The patient’s recovery was achieved through a balanced, multidisciplinary strategy that:

1. Prioritized anti-infective therapy when infection was the dominant driver,
2. Continued diuresis to optimize volume status,
3. Cautiously relaxed rate control to preserve cardiac output.

This illustrates a fundamental principle in geriatric cardiology: treatment must be dynamically adjusted based on the prevailing pathophysiology, rather than following a fixed protocol. Of course, this observation derives from a single case and requires further study.

### 3.6. Microbiological considerations and antibiotic de-escalation

The sputum culture confirmed *K pneumoniae* infection, and antimicrobial susceptibility testing provided critical guidance for antibiotic stewardship. The isolate was identified as extended-spectrum beta-lactamase (ESBL)-negative, which is a key finding. This phenotype indicates susceptibility to third-generation cephalosporins, such as ceftriaxone, while intrinsic resistance to ampicillin, ticarcillin, and macrolides is a known characteristic of *K pneumoniae* and was appropriately disregarded in the treatment selection.

Our initial empirical regimen combined moxifloxacin (to cover atypical pathogens and community-acquired pneumonia) and ceftriaxone (to cover gram-negative bacilli). When moxifloxacin was discontinued on day 5 due to neuropsychiatric adverse effects, we did not need to escalate to carbapenems. The ESBL-negative result, which became available post hoc, confirmed that ceftriaxone monotherapy was a safe and effective definitive therapy. This case illustrates a practical principle: when adverse drug reactions necessitate the withdrawal of a broad-spectrum agent, timely microbiological data (especially ESBL status) can guide a rational de-escalation strategy, preventing unnecessary use of reserve antibiotics and reducing the risk of further resistance.

### 3.7. AF in the octogenarian population: risk stratification and prognosis

This case also prompts consideration of the broader implications of AF in the very elderly. Recent studies have highlighted the prognostic significance of AF and its subclinical precursors, such as atrial high-rate episodes (AHREs), in octogenarians. For instance, Hayiroglu et al demonstrated that the occurrence of AHREs and AF following dual-chamber pacemaker implantation is a significant independent predictor of long-term mortality in patients aged 80 and older, with LVEF, left atrial diameter, and serum albumin levels also emerging as key prognostic factors.^[13]^ Furthermore, malnutrition, as assessed by the Controlling Nutritional Status score, has been shown to be independently associated with an increased risk of AHREs/AF and all-cause mortality in this age group, suggesting that nutritional status is a crucial, potentially modifiable risk factor.^[14]^ These findings underscore the importance of comprehensive risk stratification that extends beyond arrhythmia management to include nutritional, functional, and geriatric assessments, particularly in frail elderly patients who are at the highest risk of adverse outcomes.

### 3.8. Strengths and limitations

This case report offers several strengths that enhance its educational and clinical value. First, it provides a richly documented example of the dynamic interplay between new-onset AF, HFpEF, and acute infection, a common yet diagnostically challenging scenario in geriatric practice. The use of serial bedside ultrasound to track the evolution of pleural effusion offers a novel and practical teaching tool: it visually captures the synergistic effects of infection and HF, reinforcing the concept of mixed respiratory failure in a way that static imaging cannot. Second, the case underscores the importance of individualized rate control targets, challenging rigid adherence to guideline-derived numbers in hemodynamically vulnerable patients. Third, the detailed timeline of clinical events, biomarkers, and imaging findings (as summarized in Table 1) provides a transparent and reproducible framework for clinicians encountering similar clinical dilemmas.

However, several limitations should be acknowledged. As a single case report, the findings are not generalizable and cannot establish causality. Consistent with the case report format, all conclusions are hypothesis-generating and should not be interpreted as causal. The observed association between rate control, infection, and clinical deterioration, while clinically compelling, remains hypothesis-generating rather than definitive. Additionally, the absence of certain diagnostic data (such as invasive hemodynamic monitoring, serial lactate measurements, or more comprehensive inflammatory markers) limits our ability to fully characterize the pathophysiological mechanisms at play. The neuropsychiatric event attributed to moxifloxacin, while managed effectively, was not formally assessed using a standardized causality scoring system (e.g., Naranjo scale), which would have strengthened the adverse event reporting. Finally, while the patient provided informed consent, the retrospective nature of the report introduces potential selection and recall bias.

Despite these limitations, this case contributes meaningfully to the literature by illustrating a real-world clinical scenario where diagnostic vigilance, dynamic assessment, and therapeutic flexibility were essential to achieving a favorable outcome. It also highlights several testable hypotheses for future research; for instance, whether ultrasound-guided decongestion strategies improve outcomes in mixed cardiopulmonary failure, or whether lenient rate control during acute illness reduces the risk of decompensation in HFpEF patients with new-onset AF.

## 4. Conclusion

This case provides a clinically instructive example of the complex interplay between new-onset AF, HFpEF, and acute pulmonary infection in an elderly patient. It delivers several take-home messages for practicing clinicians:

1. Infection can masquerade as HF exacerbation in geriatric patients, necessitating a high index of suspicion and dynamic reassessment.
2. Respiratory failure in this setting is often mixed, resulting from synergistic cardiogenic and infectious mechanisms, best appreciated through serial imaging and biomarker trends.
3. Heart rate targets should be individualized, with attention to perfusion rather than rigid numerical goals, particularly during acute stress. However, the optimal approach in the setting of concurrent infection remains uncertain and requires further investigation.
4. Holistic, adaptive management (addressing the dominant pathology at each phase) is essential for optimizing outcomes. This includes meticulous monitoring of electrolytes and acid-base status, as even mild disturbances can have significant clinical implications in this vulnerable population.

By illustrating these principles through a well-documented clinical course, this case offers hypothesis-generating insights for internists, geriatricians, cardiologists, intensivists, and trainees navigating the complex acute care of elderly patients.

## 5. Patient perspective

The patient and her family expressed satisfaction with the treatment outcomes and overall hospital experience. When approached during one of the routine monthly follow-ups for informed consent to publish this case report, the patient authorized her daughter to sign the consent form on her behalf, reflecting her trust in the clinical team and her willingness to contribute to medical education.

Notably, the patient’s confidence in the care received was further demonstrated when she voluntarily returned to our department for readmission in February 2026, following an episode of symptom exacerbation triggered by bilateral lung infection (Fig. S1, Supplemental Digital Content 2). She was successfully treated again and has since been discharged in stable condition. This continuity of care underscores the patient’s positive perception of her treatment experience and the therapeutic alliance established during the index hospitalization.

## Acknowledgements

We appreciate the patient’s trust in us and her support for clinical work.





## Author contributions

**Data curation:** Xin Zhang.

**Investigation:** Xiaojun Yang.

**Formal analysis:** Haodong Huang.

**Funding acquisition:** Yulong Huang, Hong Yan.

**Project administration:** Fangting Wang, Yujing Chen.

**Resources:** Yulong Huang.

**Validation:** Hong Yan.

**Visualization:** Zhitong Deng.

**Writing – original draft:** Yulong Huang.

**Writing – review & editing:** Xiaojun Yang.
