# Supplementary material for: Walking the tightrope: Mixed respiratory failure exacerbated by heart rate control and concurrent pneumonia in an elderly patient with new-onset atrial fibrillation: A case report
Source: Medicine (Baltimore). 2026 Aug 21;105(34):e50388. doi: 10.1097/MD.0000000000050388 (PMC13502879; doi:10.1097/MD.0000000000050388)
Supplement: Supplementary file 1 [file medi-105-e50388-s001.doc]

****Table_S1. Antimicrobial susceptibility testing results of Klebsiella pneumoniae isolated from sputum culture.****

| **Bacterial Name** | **Antibiotic Name** | **Result** |
| --- | --- | --- |
| *Klebsiella pneumoniae* | Extended-spectrum beta-lactamase (ESBL) | Negative |
| *Klebsiella pneumoniae* | Amikacin | Susceptible |
| *Klebsiella pneumoniae* | Ertapenem | Susceptible |
| *Klebsiella pneumoniae* | Imipenem | Susceptible |
| *Klebsiella pneumoniae* | Cefuroxime sodium | Susceptible |
| *Klebsiella pneumoniae* | Cefoxitin | Susceptible |
| *Klebsiella pneumoniae* | Ceftriaxone | Susceptible |
| *Klebsiella pneumoniae* | Cefepime | Susceptible |
| *Klebsiella pneumoniae* | Cefoperazone/sulbactam | Susceptible |
| *Klebsiella pneumoniae* | Trimethoprim/sulfamethoxazole (Co‑trimoxazole) | Susceptible |
| *Klebsiella pneumoniae* | Levofloxacin | Susceptible |
| *Klebsiella pneumoniae* | Tigecycline | Susceptible |
| *Klebsiella pneumoniae* | Amoxicillin/clavulanic acid | Susceptible |
| *Klebsiella pneumoniae* | Piperacillin/tazobactam | Susceptible |
| *Klebsiella pneumoniae* | Ceftazidime | Susceptible |
| *Klebsiella pneumoniae* | Cefuroxime axetil | Susceptible |

**Specimen:** Sputum
**Multidrug-resistant organisms:** None
